# Old divergences in a boreal bird supports long-term survival through the Ice Ages

**DOI:** 10.1186/1471-2148-10-35

**Published:** 2010-02-04

**Authors:** Takema Saitoh, Per Alström, Isao Nishiumi, Yoshimitsu Shigeta, Dawn Williams, Urban Olsson, Keisuke Ueda

**Affiliations:** 1 Department of Life Science, Rikkyo University, 3-34-1 Nishi-ikebukuro, Toshima-ku, Tokyo 171-8501, Japan; 2 Swedish Species Information Centre, Swedish University of Agricultural Sciences, Box 7007, SE-750 07 Uppsala, Sweden; 3 Swedish Museum of Natural History, Box 50007, SE-104 05 Stockholm, Sweden; 4 Department of Zoology, National Museum of Nature and Science, 3-23-1 Hyakunin-cho, Shinjuku-ku, Tokyo 169-0073, Japan; 5 Yamashina Institute for Ornithology, 115 Konoyama, Abiko, Chiba 270-1145, Japan; 6 Department of Zoology, University of Göteborg, Box 463, 405 30 Göteborg, Sweden; 7 Current address: Yamashina Institute for Ornithology, 115 Konoyama, Abiko, Chiba 270-1145, Japan

## Abstract

**Background:**

Unlike northern Europe and most of northern North America, the Eastern Palearctic and the northwesternmost tip of North America are believed to have been almost unglaciated during the Quarternary glacial periods. This could have facilitated long-term survival of many organisms in that area. To evaluate this, we studied the phylogeography in east Asia and Alaska of a boreal migratory passerine bird, the Arctic Warbler *Phylloscopus borealis*, and compared our results with published data on especially North American species.

**Results:**

In a sample of 113 individuals from 18 populations we identified 42 haplotypes of the mitochondrial cytochrome *b *gene, which separated into three clades: A - Alaska and mainland Eurasia (except Kamchatka); B - Kamchatka, Sakhalin and Hokkaido; and C - Honshu, Shikoku and Kyushu (i.e. Japan except Hokkaido). The oldest split among these clades, between A/B and C, is estimated to have taken place sometime between the mid Pliocene and early Pleistocene, and the second divergence, between clades A and B, in the early to mid Pleistocene. Within all of the three main clades, there are signs of population expansion.

**Conclusions:**

The Arctic Warbler separated into three main clades in close succession around the Pliocene/Pleistocene border, with the two northern clades diverging last. All three clades probably experienced population bottlenecks during the Pleistocene as a result of range shifts and contractions, but nevertheless survived and maintained their integrities. Several other clades of Northeastern Palearctic birds are noted to have diversified during the Pliocene. In contrast, avian species or phylogroups presently occupying formerly glaciated North American ground are generally younger. The differences between these regions could be due to slower speciation rates in the Eastern Palearctic due to less fragmentation of forest habitats during glacial periods, or to longer survival of Eastern Palearctic clades as a result of less severe conditions in that region compared to northern North America. Several other Palearctic organisms show concordant biogeographical patterns to that of the Arctic Warbler, indicating common causes of their diversifications.

## Background

In recent years, substantial knowledge accumulated on the genetic consequences of the climatic oscillations in the Quaternary for European and North American species [e.g. [1-4]]. Since the ice sheets repeatedly spread considerably southward on both continents during glacial periods (to 52°N in Europe and 40°N in North America at the last glacial maximum, 23-18 kya; [3]), boreal and temperate biota were repeatedly pushed southward into isolated refugia and subsequently recolonized northward again. As a result, shallow genetic divergence [5-8] and low genetic diversity [1,9,10] are typical of various taxa in northern regions. In contrast, relatively deep DNA divergences [7,11] and higher genetic diversity [1] occur in many taxa inhabiting temperate refugial areas in Europe and North America, suggesting survival of these populations over several glacial periods. Since the Eastern Palearctic and northwesternmost tip of North America are considered to have remained largely ice-free throughout the Quaternary [12-14] (but see [15] for a different opinion), deep DNA divergences and high genetic diversity would also be expected in that region. However, even in the absence of ice sheets, the habitats in that region are believed to have been much affected by the climatic oscillations, and this is likely to have affected the distributions and consequently also the population dynamics of many animals.

Several studies have dealt with the phylogeography of widespread northern Palearctic animals, including mammals such as badger *Meles meles *[16], hares *Lepus *spp. [17], and various rodents [18-23], as well as birds including ducks [24], shorebirds[25,26], woodpeckers [27,28], and several species of passerines [26,29-41]. Most of these studies have reported little or no divergence over large areas of the northern Palearctic, although some divergent clades, mostly dated to the Pleistocene, have been found in some species. For example, a southeastern Palearctic clade has been noted in several birds and mammals and also in an amphibian and two ants, suggesting common causes of their divergences (reviewed in [22]). Hewitt et al [3] concluded that some Arctic species have shallow genetic clades with coinciding geographical boundaries. Some phylogeographical studies of birds have dealt with groups of closely related species with extensive north-south distributions from the taiga to temperate forests in central China and the Himalayas, and some of these have found relatively deep divergences between boreal and more southern taxa, which date back to the Pliocene or early Pleistocene [42-44].

**Figure 1:**
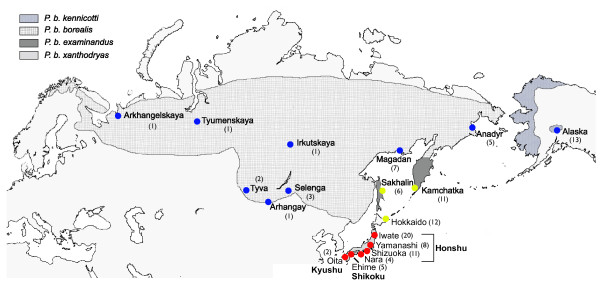
**Distribution of the Arctic Warbler *Phylloscopus borealis***. Map based on [46], with the ranges of the subspecies according to [49] (Sakhalin population not studied by [49], but thought to belong to *examinandus*). The sampling sites are marked, with the sample sizes indicated by numbers. Blue circles represent clade A, yellow circles clade B, and red circles clade C.

The Arctic Warbler *Phylloscopus borealis *is a small insectivorous passerine, which breeds in the boreal forests from northern Scandinavia through Siberia to Alaska, south to northern Mongolia, Russian Far East, northeasternmost China and Japan [45,46] (Fig. 1). All populations winter in Southeast Asia, Indonesia and the Philippines [45,46]. This is the only species in the large genus *Phylloscopus *that breeds in North America [45]. Three to six morphologically subtly different subspecies are generally recognised, with much disagreement among authors [45-51] (Fig. 1). In the breeding season, it inhabits both coniferous, mixed and broad-leaved forest, and also bushes (e.g. *Salix*) on the tundra and above the tree limit; in Japan it breeds in mountains up to 2500 m [45-47,52]. A recent paper [53] analysed mitochondrial ND2 sequence variation across a large part of the species' continental range (but not including Japan), and found little divergence, except between Kamchatka/Sakhalin and the rest of the range, concordant with results from several other studies on other species as noted above [22]. The same study [53] also found the haplotypes from northeast Siberia and Alaska to form a clade separated from the haplotypes from south Siberia to northeast Europe. Moreover, the nucleotide diversity was highest in south Siberia/northeast Europe and lowest in Alaska, with northeast Siberia having intermediate values. It was concluded that the direction of postglacial colonization was likely from south Siberia towards the northwest and northeast, with Alaska being colonized in a stepping-stone pattern.

In this paper, we conduct a more extensive analysis of variation in mitochondrial DNA (1-3.2 kbp) from 18 populations of the Arctic Warbler from across the species' range, including denser sampling from the previously poorly sampled Kamchatka and Sakhalin and the formerly unsampled Japan, in order to examine: (1) the phylogeographic structure compared with other boreal species, with the specific hypothesis that the Eastern Palearctic/Northwesternmost Nearctic populations might show deep divergences as a result of long-time survival in a region which has been less affected by ice during the Pleistocene than other parts of the Holarctic; (2) whether the genetic diversity is high throughout this part of the range, as in many species inhabiting unglaciated temperate refugial regions in Europe and North America; and (3) whether latitudinal trends in genetic diversity and signs of population expansions are lacking, unlike in European and North American species inhabiting formerly glaciated areas.

## Results

### Phylogeny and divergence times

Forty-two cytochrome *b *haplotypes were identified among the 113 individuals from 18 populations (Table 1, Additional file 1) based on 99 polymorphic sites (91 transitions, 8 transversions). According to the AMOVA, 3.2% of the variance is attributable to within-population variation and 96.8% to among-population variation.

**Table 1:** Frequency of cytochrome *b *haplotypes in all Arctic Warblers sampled

	Population														
	
Haplotype	ALA	ANA	MAG	CSH	WSH	KAM	SAK	HOK	IWA	YAM	SHI	NAR	EHI	OIT	*N*
A1	7														7
A2	5														5
A3	1		1												2
A4		1													1
A5		2													2
A6		1													1
A7		1													1
A8			1												1
A9			2												2
A10			1												1
A11			1												1
A12			1												1
A13					1										1
A14					1										1
A15				3											3
A16				1											1
A17				1											1
A18				1											1
A19				1											1
B1						2	1								3
B2						5	1								6
B3						1		10							11
B4						1									1
B5						1									1
B6						1									1
B7							1	1							2
B8								1							1
B9							1								1
B10							1								1
B11							1								1
C1									19	4	2		2		27
C2										1					1
C3											5				5
C4											1				1
C5										1					1
C6										1					1
C7												2			2
C8													1		1
C9												2			2
C10										1					1
C11									1		3		2	1	7
C12														1	1
*N*	13	5	7	7	2	11	6	12	20	8	11	4	5	2	113

N = total sample size. ALA = Alaska; ANA = Anadyr; MAG = Magadan; CSH = central Siberia (Irkutskaya, Selenga, Arhangay, Tyva); WSH = western Siberia (Arkhangelskaya, Tyumenskaya); KAM = Kamchatka; SAK = Sakhalin; HOK = Hokkaido; IWA = Iwate; YAM = Yamanashi; SHI = Shizuoka; NAR = Nara; EHI = Ehime; OIT = Oita. See Figure 1.

**Figure 2:**
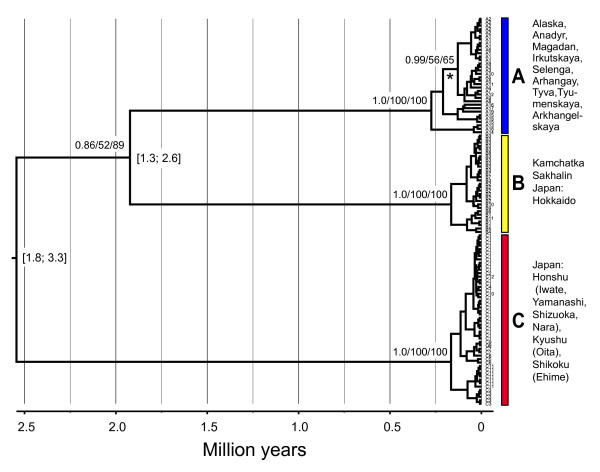
**Mitochondrial cytochrome *b *gene tree**. Dated gene tree for all cytochrome *b *sequences (including identical haplotypes), estimated by Bayesian inference using the GTR + Γ model, a fixed clock rate of 0.0105 per lineage/million years and a coalescent expansion growth model. Values in square brackets are 95% highest posterior density intervals for the node ages. Support values are given above the nodes (>0.5/50%) in the order, from left to right, posterior probability, maximum likelihood bootstrap (1000 replicates) and parsimony bootstrap (1000 replicates); support for minor clades within the three main clades are not indicated, except for a clade comprising samples from northeast Siberia and Alaska (marked by an asterisk). Clades discussed in the text are indicated by A, B and C.

In the cytochrome *b *tree comprising all samples (Fig. 2) the Arctic Warbler is divided into three main, strongly supported clades (A, B and C). These represent separate geographical locations: clade A - Alaska and mainland Eurasia (except Kamchatka); clade B - Kamchatka, Sakhalin and Hokkaido; and clade C - Honshu, Shikoku and Kyushu (cf. Fig. 1). In the BEAST and bootstrap analyses, clades A and B are inferred to be sisters, although with low support (Fig. 2), whereas in the MrBayes analysis (not shown) clades A and C are inferred to be sisters, although with effectively no support (0.55). Within clades A, B and C there is no correlation between the inferred relationships of the sequences and their geographical locations, except that the sequences from northeast Siberia and Alaska form a well supported clade both in the BEAST (Fig. 2) and MrBayes analyses (0.93; not shown) (but with insignificant support in the bootstrap analyses).

**Figure 3:**
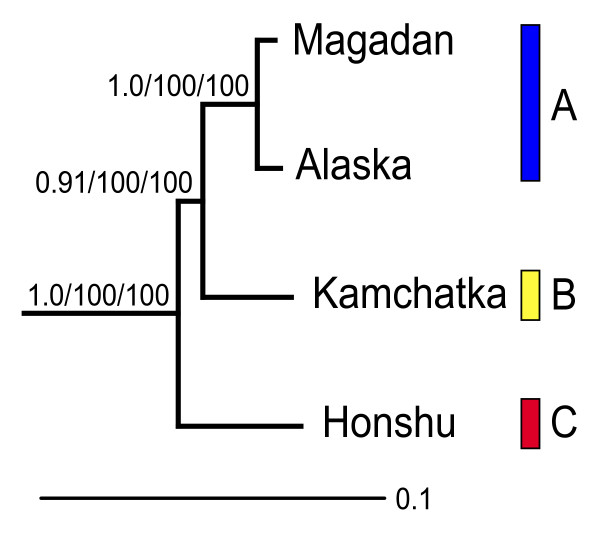
**Relationships among the three main clades**. Relationships of representatives from each of the three main clades in Figure 2, estimated by Bayesian inference of concatenated mitochondrial ND5, cytochrome *b*, control region, tRNA-Pro, ND6, tRNA-Glu, tRNA-Phe and 12S sequences (3.2 kbp), under the HKY + I model. Support values at the nodes are, from left to right, posterior probability, maximum likelihood bootstrap and parsimony bootstrap. Clade names (A, B, C) same as in Figure 2.

In the tree estimated from the concatenated ND5-cytochrome *b*-control region-ND6-12S-tRNA sequences (Fig. 3) the samples representing clades A and B are recovered as sisters, with 100% parsimony and maximum likelihood bootstrap support and somewhat lower posterior probability.

The estimated ages of the main clades differ among the analyses. In the analyses with a fixed clock rate of 2.1% per million years (MY) the mean age of the split between clades A/B and C is inferred to be 2.5 or 3.0 MY, i.e. in the early Pleistocene or late Pliocene, respectively, and between clades A and B 1.9 or 2.3 MY, i.e. in the early Pleistocene (Table 2, Figs. 2, 5). The analyses employing a lognormal uncorrelated relaxed clock with a fixed mean rate result in ages with large differences in means (A/B-C: 2.1 and 3.6 MY, respectively) and confidence intervals of up to 4.4 MY (not shown). We conclude that a relaxed clock prior is unsuitable for our data in the absence of independent information, such as fossils, that can help us define a strong prior on the time to most recent common ancestor.

**Table 2:** Estimated ages of the main Arctic Warbler clades

Clades	All samples	Major clades
A--B	1.9 [1.3--2.6]	2.3 [1.5--3.0]
A/B--C	2.5 [1.8--3.3]	3.0 [2.1--3.8]

Estimated by Bayesian inference using the GTR + Γ model and a fixed clock rate of 0.0105 per lineage/million years, and a coalescent expansion growth model (All samples) or birth-death model (Major clades). Values in square brackets are 95% highest posterior density intervals.

The estimated mean ages of the deepest splits within clades A, B and C are approximately 0.17-0.28 MYA (Fig. 2), although most divergences are much shallower (and many haplotypes are identical).

### Population genetics and demography

**Figure 4:**
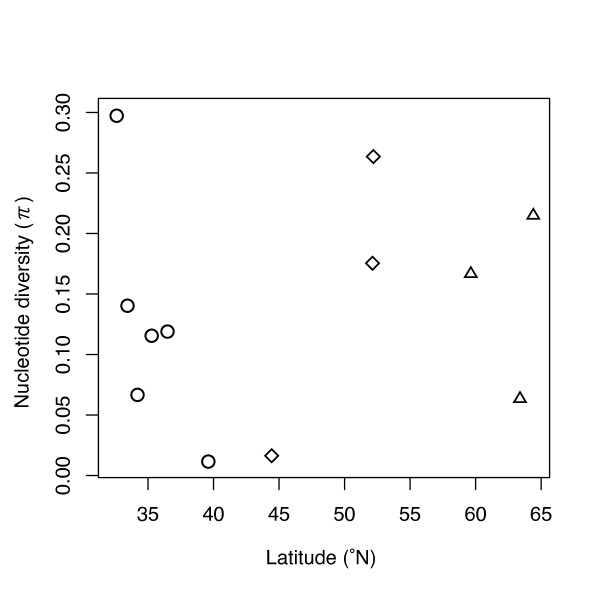
**Nucleotide diversities (π) as a function of latitude**. Triangles denote populations belonging to Clade A, rhomboids Clade B, and circles Clade C.

Within each clade, some populations share haplotypes: e.g., haplotype A3 is found in Alaska and Magadan (clade A); haplotypes B1 and B2 in Kamchatka and Sakhalin, B3 in Kamchatka and Hokkaido, and B7 in Sakhalin and Hokkaido (clade B); and haplotypes C1 in Iwate, Yamanashi, Shizuoka and Ehime (clade C) (Table 1). Haplotype diversity (*h*) and nucleotide diversity (*π*) are shown in Table 3. Overall haplotype diversity (0.921) and nucleotide diversity (2.97%) are high. However, within the three main clades the mean diversity estimates are lower, especially the nucleotide diversity (*h*: clade A, 0.887; clade B, 0.818; clade C, 0.687; *π*: clade A, 0.29%; clade B, 0.15%; clade C, 0.10%), and the differences among clades A-C are not significant (ANOVA:*h, F *= 0.65, *p *= 0.54; *π*, *F *= 0.25, *p *= 0.78). There are no latitudinal trends in haplotype and nucleotide diversity within the three main clades (*h*, Spearman rank correlation: clade A, *rs *= -0.5, n = 3, *p *= 1.00; clade B, *rs *= 1.0, n = 3, *p *= 0.33; clade C, *rs *= -0.77, n = 6, *p *= 0.10; *π*, Spearman rank correlation: clade A, *rs *= 0.5, n = 3, *p *= 1.00; clade B, *rs *= 1.0, n = 3, *p *= 0.33; clade C, *rs *= -0.6, n = 6, *p *= 0.35; Fig. 4). However, diversity estimates in peripheral populations within the main clades are relatively low (Alaska, clade A; Hokkaido, clade B; Iwate, clade C; Table 3).

**Table 3:** Sample size (N), diversity estimates and statistics for evidence of population expansion

Clade/population	*N*	No. of haplotypes	Haplotype diversity (*h*)	Nucleotide diversity (*π*) (%)	Tajima's *D*	Fu's *F S*
**clade A**	25	19	0.887 ± 0.04	0.290 ± 0.178	-1.435 (*p *= 0.07)	-11.983 (*p *= 0.0000**)
Alaska	13	3	0.603	0.066		
Anadyr	5	4	0.9	0.217		
Magadan	7	5	0.952	0.169		
C Siberia	7	5	0.857	0.273		
W Siberia	2	2	1	0.099		
**clade B**	29	15	0.818 ± 0.0564	0.146 ± 0.101	-1.345 (*p *= 0.075)	-6.180 (*p *= 0.0000**)
Kamchatka	11	6	0.8	0.176		
Sakhalin	6	6	1	0.264		
Hokkaido	12	3	0.318	0.017		
**clade C**	50	18	0.687 ± 0.067	0.104 ± 0.078	-1.799 (*p *= 0.016*)	-7.993 (*p *= 0.0000**)
Iwate	20	2	0,1	0,01		
Yamanashi	8	5	0.786	0.117		
Shizuoka	11	4	0,746	0,115		
Nara	4	2	0,667	0,066		
Ehime	5	3	0,8	0,138		
Oita	2	2	1	0,3		

See Table 1 for localities included in Central Siberia and West Siberia, respectively, and Figure 1 for geographical locations.

The pairwise *F*st values for the localities for which our sample sizes are ≥7 are shown in Table 4. *F*st values are higher between clades A, B and C (0.946-0.998) than within these main clades (0.166-0.703). The highest value is between Hokkaido (clade B) and Iwate (clade C), which are only approximately 600 km apart. The *F*st value between Alaska and Magadan is 0.499, and significantly different, despite the sharing of one haplotype. Combining samples from localities with small sample sizes with geographically adjacent localities (e.g. west and central Siberia or Anadyr and Magadan) yield very similar results.

**Table 4:** Pairwise Fst values among different populations of the Arctic Warbler.

	Population	1	2	3	4	5	6	7	8
1	Alaska	--							
2	Magadan	0.499	--						
3	Central Siberia	0.703	0.495	--					
4	Kamchatka	**0.973**	**0.959**	**0.946**	--				
5	Hokkaido	**0.990**	**0.983**	**0.973**	0.293	--			
6	Iwate	*0.993*	*0.989*	*0.984*	***0.987***	***0.998***	--		
7	Yamanashi	*0.982*	*0.969*	*0.959*	***0.971***	***0.989***	0.166	--	
8	Shizuoka	*0.981*	*0.971*	*0.962*	***0.972***	***0.988***	0.332	0.189	--

Only populations with sample sizes of ≥7 are included. The different styles (bold; italic; bold italic) represent pairwise *F*st values among the three main clades (A, B and C) in Figures 2 and 3. All comparisons are significant (*p *< 0.05).

The Tajima's *D *values of all three major clades are negative (Table 3), which suggests sudden population expansions, although only that of Clade C is significant. In addition, Fu's *Fs *are negative, with significant *p-*values for all three main clades (Table 3), again suggesting past population expansions.

## Discussion

### Phylogeny, divergence times and comparison with North America

The three main clades are highly divergent and well supported, and are estimated to have diverged around the Pliocene/Pleistocene border. The combined sequence data provide reasonably strong support for a sister relationship between the two northerly distributed clades (A and B).

**Figure 5:**
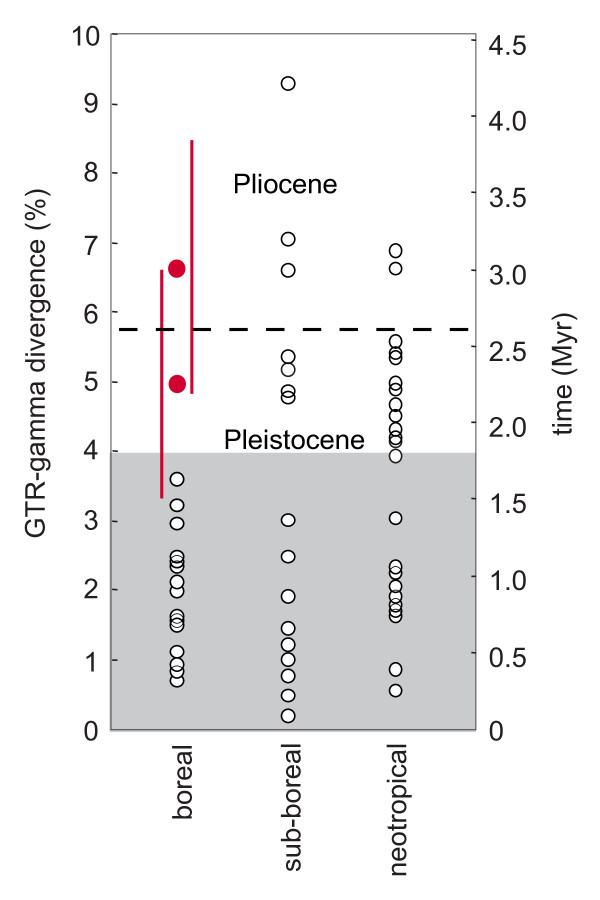
**Dates of divergence for the main clades of Arctic Warblers compared to divergences among Nearctic and Neotropical species belonging to superspecies**. Modified from [7]. Open dots represent genetic distances (GTR+Γ corrected) and approximate dates of coalescence events between closely related New World species belonging to superspecies in boreal forest, sub-boreal and neotropical lowland avifaunas. Red dots symbolize the means of the estimated ages of the Arctic Warbler clades (A--B, A/B--C), and the red vertical bars represent the 95% highest posterior density intervals (based on one sequence per main clade, GTR+Γ, fixed clock rate 0.0105/lineage/MY and birth-death model). The Pleistocene as defined by [7] is indicated by the shaded area, whereas the limit of the Pleistocene as used here (International Union of Geological Sciences) is indicated by the dashed line.

Weir & Schluter [7] found, based on an extensive survey of New World species, that 100% of boreal North American members of superspecies coalesced during the Pleistocene, in contrast to 56% of sub-boreal and 46% of tropical superspecies. A similar latitudinal trend of divergence times has been found in North American fishes: clades occupying formerly glaciated areas are generally of Pleistocene origin [54,55], whereas more southerly distributed clades usually predate the Pleistocene [56]. The separation of the three main Arctic Warbler clades in the Pliocene or early Pleistocene is older than the divergences of closely related species from formerly ice-covered boreal regions of North America, but in agreement with species pairs from temperate North American and Neotropical regions [7] (Fig. 5) (note that [7] used different limits of the Pleistocene than done here, see Fig. 5).

Several other studies suggest that sister species of boreal Eastern Palearctic forest birds might be on average older than their New World counterparts. Boreal Eastern Palearctic sister species of *Phylloscopus *warblers are estimated to have diverged between mid-Pliocene and mid-Pleistocene [57]. The widely distributed boreal *Parus montanus *separated from the Eastern Palearctic *Parus affinis *around the Pliocene-Pleistocene border [58]. In a clade of boreal mainly Eastern Palearctic *Emberiza *buntings, uncorrected cytochrome *b *divergences are 5.1-8.3% [59], indicating separation during the Pliocene (assuming 2.1% divergence per million years; [60]). Two species of *Erithacus *robins, one endemic to Japan and one occurring in Japan and on Sakhalin, are estimated to have separated from their mainland relatives 1.5-5.2 Mya, during the Pliocene or early Pleistocene [61]. In contrast, Nylander *et al*. [62] inferred that most Palearctic thrush *Turdus *spp. sister species, several of which have boreal Eastern Palearctic distributions, separated in the mid to late Pleistocene (although the Southeast Palearctic *T. mupinensis *was inferred to have separated from a common ancestor with an African species during the early Pliocene).

The generally older ages of boreal sister species in the Eastern Palearctic than in North America could be due to lower recent speciation rates or lower extinction rates in the Eastern Palearctic (or a combination). Weir & Schluter [7] concluded that the fragmentation of forests by ice sheets during the Pleistocene was the main cause of the elevated rates of diversification of forest birds in boreal regions compared to sub-boreal and tropical New World regions. It is possible that forest habitats were less fragmented in the Eastern Palearctic than in North America during the Pleistocene (but see below), causing less subdivision of forest bird populations and hence less divergence. Alternatively, the less severe conditions in the Eastern Palearctic than in northern North America, e.g. [12-14,63] might have facilitated long-term survival of already separated lineages.

### Distributional patterns

The three major Arctic Warbler lineages have apparently maintained their integrity through several successive glaciations, with little or no mixing, despite likely shifts and contractions of their breeding ranges and shared winter distributions. The divergence between clade C and the common ancestor of clades A and B is here postulated to have occurred between 2.5 and 3.0 mya. This coincides with the climate crash associated with the final closure of the Panama Isthmus at 2.7 MYA. Northern Hemisphere ice sheets expanded, and there was a global tendency toward a more arid climate, causing among other things decreasing and fragmented forest cover [64-66]. During the last glacial maximum, 14 000-26 000 years ago, the northwesternmost part of the present range of the Arctic Warbler was glaciated, while the rest of northern Eurasia and northwesternmost North America was arid and tree-less, with polar desert or steppe-tundra conditions at least in higher-elevation areas, e.g. [12-14,63,67]. Parts of China and Japan are believed to have been forested [68-75]. Accordingly, the conditions were probably unsuitable for Arctic Warblers in much of its current range. However, since the Arctic Warbler can thrive in scrub on tundra further north than practically all other warblers [45-47,52], it is possible that it could have occurred at least locally within part of its present range. It seems probable that it could have remained in Japan throughout the glacial periods. Based on the current breeding distributions and the fact that all Arctic Warblers winter in Southeast Asia, the Philippines and Indonesia [45], it seems likely that all three major lineages have survived the glaciations in eastern Asia. Reeves *et al*. [53] found evidence of gene flow in continental Eurasian/Alaskan populations of Arctic Warblers (corresponding to clade A of the present study) from south Siberia towards the northwest and northeast, and concluded that this pattern probably indicates the routes of postglacial expansion.

Although we do not know for sure in which areas the three Arctic Warbler clades diverged, the combined sequence data strongly suggest that first a lineage comprising the two northern clades (A and B) separated from a common ancestor with the southern clade (C), whose distribution probably included Japan, and later the two northern clades separated from each other. This scenario makes sense also from a geographical point of view, since the ancestry of the clade including the Arctic Warbler is inferred to have been somewhat further south [57]. The very short internode leading up to the clade comprising A and B indicates that all three lineages separated close in time. A similar "simultaneous" burst of lineage splittings has been suggested for a clade of mainly Eastern Palearctic boreal *Emberiza *buntings [59].

Several species groups and phylogroups of birds and mammals have distributional patterns that agree fairly well with that of the Arctic Warbler, suggesting that geographical barriers leading to vicariant divergence of populations have existed in the past between, on the one hand, much of the northern Palearctic and, on the other hand, Russian Far East (including Sakhalin and southern Kamchatka) and Japan, and on a finer scale within the latter area, between Sakhalin/southern Kamchatka/Hokkaido and the rest of Japan. Some of these divergences may have been caused by Pleistocene climatic oscillations, while others are apparently older and must have other causes. The importance of the Tsugaru strait, "Blakiston's line", between Hokkaido and Honshu as a biogeographic division line in various taxa of animals and plants has been stressed by [76-78]. Several of the birds breeding in Japan and, in some cases, also on Sakhalin and southern Kamchatka have their closest relatives in the boreal forests on the mainland [29,57,59,61,62,79]. Three of these (*Emberiza variabilis*, *Locustella amnicola *and *Phylloscopus borealoides*) differ from their respective sister species by c. 3.4-4.4% in cytochrome *b *(uncorrected p; P. Alström & U. Olsson, unpublished). This is similar to the differences among the three main Arctic Warbler clades (3.8-5.1%, uncorrected p), suggesting common causes of their divergences. In addition, several bird and mammal species show relatively deep divergences between a clade covering much of the northern Palearctic and a clade from the Russian Far East (sometimes including Sakhalin and southern Kamchatka) and in some cases Japan [22,23,28,33,34]. However, these divergences are of more recent origin than the Arctic Warbler clades, and probably all date to the Pleistocene.

Despite apparent historical range shifs, it is possible that the three main Arctic Warbler clades have diverged in complete geographical isolation. There is presently no known geographical overlap between them, although the parts of Kamchatka and continental Russian Far East where this might occur are unsampled. The present study indicates significant divergence between some geographically closely situated localities within the main clades, even in the absence of apparent geographical barriers. Reeves *et al*. [53] also reported significant isolation-by-distance in Arctic Warblers in continental Eurasia/Alaska. Presumably, the natal philopatry is strong and the innate migration routes strictly adhered to, which has been suggested to be at least part of the explanation for the maintenance of geographically neighbouring clades in the boreal migratory Willow Warbler *Phylloscopus trochilus *[80], Greenish Warbler *Phylloscous trochiloides *complex [81] and Dunlin *Calidris alpina *[25].

### Population expansions

Within each of the three main clades, the nucleotide diversity is low (mean π = 0.10-0.29%), especially in the marginal populations from Iwate (clade C), Hokkaido (clade B) and Alaska (clade A) (π = 0.010-0.066), and neutrality tests indicate sudden population expansion in each of these clades. These results suggest that each clade has suffered from past bottlenecks, and that the range of each of them has expanded in more recent times. There is observational data supporting recent expansion in at least one area: the Arctic Warbler was only recently confirmed to breed in Hokkaido, on the Shiretoko peninsula [82]. Reeves *et al*. [53] also found evidence of population expansion in Arctic Warblers in northeast Siberia/Alaska, while they inferred more stable populations in south and west Siberia and north Europe. Although they did not find any signature of population expansion in the west Siberian and European populations, these populations must have expanded their ranges considerably during the Holocene, since their present breeding areas were coverd by ice during the latest glaciation. Reeves *et al*. [53] further deduced that Beringia was likely colonized in two steps, first from south Siberia to northeast Russia and then, after a delay, across the Bering Strait to Alaska.

A similar pattern of post-Pleistocene population expansion has been found in another forest bird, the Great Tit *Parus major *complex. The northern *major *(Europe to northern Russian Far East) and eastern *minor *(China, Japan, Korean pensinsula, southern Russian Far East) groups show signs of population expansion, unlike the Central Asian *bokharensis *and South Asian *cinereus *groups [32]. It is most likely that the northern *major *group was forced to retreat south to escape from the advancing ice during the glacial periods, and that at the same time the east Asian *minor *group probably also had to reduce its range due to habitat changes resulting from the colder and drier climate. Evidence of population expansion has been found in several other widely distributed boreal forest taxa, including seven more birds, two rodents, a flying squirrel, a newt and two ants (reviewed in [22,26]).

The results suggest that although the Eastern Palearctic may have been almost free of ice during the Pleistocene, population bottlenecks and subsequent expansions have nevertheless occurred in that region because of forest dynamics.

## Conclusions

The three Arctic Warbler clades are estimated to have diverged in close succession during the latter part of the Pliocene to early part of the Pleistocene, and although all of them experienced population bottlenecks during the Pleistocene, they nevertheless survived and maintained their respective integrity. Suitable breeding habitats likely existed in east Asia, probably at least partly within the present breeding ranges of these lineages, e.g. Japan. Several other clades of Northeastern Palearctic forest birds are noted to have diversified in the late Pliocene. This pattern differs from that of North American boreal forest clades that occur on formerly glaciated ground, and which are generally of Pleistocene origin. The differences between these regions could be due to slower speciation rates in the Eastern Palearctic due to less fragmentation of forest habitats during glacial periods, or to longer survival of Eastern Palearctic clades as a result of less severe conditions in that region compared to northern North America. Several other Palearctic organisms show concordant biogeographical patterns to that of the Arctic Warbler, indicating common causes of their diversifications.

## Methods

### Sampling and laboratory methods

A total of 113 Arctic Warbler samples were obtained during the breeding season from 18 localities across the entire breeding range (Fig. 1, Additional file 1). Samples were also obtained from two of the closest relatives of Arctic Warbler, Large-billed Leaf Warbler *P. magnirostris *and Sakhalin Leaf Warbler *P. borealoides*, as well as two more distantly related *Phylloscopus *species, Two-barred Warbler *P. plumbeitarsus *and Eastern Crowned Warbler *P. coronatus *[57,79].

Total DNA from blood or pectoral muscle were extracted using the standard phenol-chloroform procedure. For each individual, partial mitochondrial cytochrome *b *(1012 bp) was amplified with primers mt-F (H16065) [83] and mt-A (L14970) [84]. The PCR reactions were performed in a total volume of 35 μl using 10 ng of total DNA, 1.5 mM MgCl_2_, 0.2 mM of each dNTP, 0.4 μM of each primer, 0.5 units Ex-Taq polymerase (Takara). The amplification profile was 94°C for 3 min followed by 35 cycles of 94°C for 30 sec, 56°C for 30 sec, and 72°C for 1 min and a final extension in 72°C for 5 min, using Takara PCR Thermal Cycler MP (Takara).

For one or two individuals from each of the three main cytochrome *b *clades (see Results) and the two outgroup taxa we also amplified part of the mitochondrial ND5 gene (964 bp), and a sequence comprising part of the mitochondrial control region, tRNA-pro gene, NADH dehydrogenase subunit 6 (ND6) gene, tRNA-Glu and tRNA-Phe genes, and part of the 12S rRNA gene (hereafter CR-ND6-12S-tRNA; 1232 bp excluding gaps in the alignment). For ND5, we used primers mt-F [83] and L14080ND5P, 5'-TCAACYCAYGCMTTCTTCAAAGC-3' (modified from [85]), which amplifies approximately 2 kbp (ND5 and cytochrome *b*), and the amplification profile was 94°C for 3 min followed by 35 cycles of 94°C for 30 sec, 53°C for 30 sec, and 72°C for 1.5 min and a final extension in 72°C for 5 min. The CR-ND6-12S-tRNA region was amplified using primers, DLL3, 5'-TGATGCACTTTGACCCCATTCATGG-3' and 12SH2, 5'-AGCAACAACCAACGGTAAG-3' and amplification profile [86]. The PCR cycling parameters were 2.5 min at 95°C; 40 cycles of 30 s at 95°C, 30 s at 50°C, and 2 min at 72°C; terminated by 7 min at 72°C, and a 4°C soak. PCR products were purified using ExoSAP-IT (Amersham Bioscience).

Sequencing reactions were performed with the primers L14080ND5P, mt-A and mt-F with BigDye Terminator Cycle Sequencing FS Kit v.3.1 and run with ABI 3100-Avant sequencer (Applied Biosystems). For some samples, products were purified using EZNA cycle pure kit (Omega bio-tek), and sequencing performed by Macrogen Inc., Seoul, South Korea, using the primers DLL3, 12SH2 and DLLF2. The sequences were checked to make sure that coding regions contained no stop codons.

### Data analyses

The sequences were aligned by eye with ATGC v. 4.0.8 and GENETYX-MAC v.10.1 (GENETYX).

Phylogenetic trees were inferred using BEAST version 1.5.2 [87,88]. Xml files for analysis in BEAST were generated in BEAUti version 1.5.2 [89] using a GTR+Γ model and fixed clock rate of 0.0105 (corresponding to 1.05%/MY/lineage: [60]). A coalescent expansion growth model was used in analyses comprising all sequences of Arctic Warblers, including identical haplotypes (as suggested at http://beast.bio.ed.ac.uk/FAQ#Should_I_remove_identical_sequences.3F), but no outgroups. A birth-death model was used for datasets including one sequence per main clade in the Arctic Warbler complex (as revealed by other analyses) and outgroups. The data were also analysed employing a lognormal uncorrelated relaxed clock [90] with a fixed mean rate of 0.0105 per lineage/MY [60]. Default priors were used. 40 × 10^6 ^generations were run, sampled every 1000 generation. The MCMC output was analysed in Tracer version 1.4.1 [91] to evaluate whether valid estimates of the posterior distribution of the parameters had been obtained. The first 25% of the generations were discarded as "burn-in", well after stationarity of chain likelihood values had been established. Trees were summarized using TreeAnnotator version 1.5.2 [92], choosing "Maximum clade credibility tree" and "Mean heights", and displayed in FigTree version 1.2.3 [93]. Only the cytochrome *b *dataset was analysed in BEAST.

Phylogenetic trees were also constructed by Bayesian inference using the program MrBayes 3.1.2 [94]. Appropriate substitution models were determined based on the Akaike Information Criterion [95] and a hierarchical likelihood ratio test [96], both calculated using MrModeltest2 [97] in conjunction with PAUP* [98]. The selected model for cytochrome *b *was a general time-reversible (GTR) model [99-101] with an estimated proportion of invariant sites (I; [102]) (GTR+I), and for the concatenated ND5, cytochrome *b*, CR-ND6-12S-tRNA sequences the HKY model [103] with an estimated proportion of invariant sites (I; [102] (HKY + I). Default priors were used. Four Metropolis-coupled MCMC chains were run for 20 × 10^6 ^generations, and sampled every 1000 generations; the heating temperature was set to 0.1. Two independent analyses were run simultaneously, starting from random trees (per default). The first 25% of the generations were discarded as "burn-in", well after stationarity of chain likelihood values had been established by inspection in Tracer 1.4.1 [91] as well as in the MrBayes summary of parameters, and the posterior probability was estimated for the remaining generations. The samples from the stationary phases of the independent runs were pooled to obtain the final results.

Parsimony (MP) bootstrapping was performed in PAUP* [98]: heuristic search strategy, 1000 replicates, starting trees obtained by stepwise addition (random addition sequence, 10 replicates), TBR branch swapping. Maximum likelihood (ML) bootstrapping (1000 replicates) was performed in Treefinder [104,105] using default settings and the same models as in MrBayes.

For the cytochrome *b *data, haplotype diversity (*h*; [106], nucleotide diversity (π; [106], number of segregating (polymorphic) sites per nucleotide (θ; [107]), pairwise *F*st [108], and analyses of molecular variance (AMOVA; [109]) were calculated with Arlequin version 3.0 [110] for all individuals.

## Authors' contributions

TS and PA participated in all parts of this study. IN and KU conceived of the study, and participated in its design and coordination and helped with a first draft of the manuscript. YS participated in design of field work and in sampling. UO carried out part of the lab work and contributed to the manuscript. DW carried out part of the lab work. All authors read and approved the final manuscript.

## Supplementary Material

Additional file 1 Appendix - Taxon, sampling locality, field and museum voucher number, sequenced region(s), cytochrome *b *haplotype and GenBank accession number of all samples Click here for file
