# Supplementary material for: Old divergences in a boreal bird supports long-term survival through the Ice Ages
Source: BMC Evol Biol. 2010 Feb 4;10:35. doi: 10.1186/1471-2148-10-35 (PMC2848155; doi:10.1186/1471-2148-10-35)
Supplement: Additional file 1 — Appendix - Taxon, sampling locality, field and museum voucher number, sequenced region(s), cytochrome b haplotype and GenBank accession number of all samples [file 1471-2148-10-35-S1.DOC]

## Additional files

## Appendix - Taxon, sampling locality, field and museum voucher number, sequenced region(s), cytochrome *b* haplotype and GenBank accession number of all samples

| Appendix. |  |  |  |  |  |  |  |
| --- | --- | --- | --- | --- | --- | --- | --- |
| **Species** | **Locality** | **Coordinates** | **Field No.** | **Museum No.** | **Sequenced region** | **Cyt*b* haplotype** | **GenBank Accession No.** |
| *Phylloscopus borealis* | Alaska, USA | 63.33N, 151.0'W | AlaRCF224 |  | Cyt*b* | A1 | [GenBank: AB362435] |
|  | Alaska, USA | 63.33N, 151.0'W | AlaRCF246 |  | Cyt*b* | A2 | [GenBank: AB362436] |
|  | Alaska, USA | 63.33N, 151.0'W | AlaRCF247 |  | Cyt*b* | A1 |  |
|  | Alaska, USA | 63.33N, 151.0'W | AlaRCF248 |  | Cyt*b* | A2 |  |
|  | Alaska, USA | 63.33N, 151.0'W | AlaRCF254 |  | Cyt*b* | A2 |  |
|  | Alaska, USA | 63.33N, 151.0'W | AlaRCF259 |  | Cyt*b* | A1 |  |
|  | Alaska, USA | 63.33N, 151.0'W | AlaRCF265 |  | Cyt*b* | A1 |  |
|  | Alaska, USA | 63.33N, 151.0'W | AlaRCF299 |  | Cyt*b* | A1 |  |
|  | Alaska, USA | 63.33N, 151.0'W | AlaRCF304 |  | ND5, Cyt*b*, CR-ND6-12S-tRNA | A2 |  |
|  | Alaska, USA | 63.33N, 151.0'W | AlaAF14339 |  | Cyt*b* | A1 |  |
|  | Alaska, USA | 63.33N, 151.0'W | AlaAF14381 |  | Cyt*b* | A1 |  |
|  | Alaska, USA | 63.33N, 151.0'W | AlaAF7143 |  | Cyt*b* | A2 |  |
|  | Alaska, USA | 63.33N, 151.0'W | AlaKSW1456 |  | Cyt*b* | A3 | [GenBank: AB362437] |
|  | Anadyr, Russia | 63.35N, 176.52E | PMN-212 | NRM 20066066 | Cyt*b* | A4 | [GenBank: AB362438] |
|  | Anadyr, Russia | 66.09N, 175.45E | PMN-246 | NRM 20066096 | Cyt*b* | A5 | [GenBank: AB362439] |
|  | Anadyr, Russia | 66.09N, 175.45E | PMN-247 | NRM 20066097 | Cyt*b* | A5 |  |
|  | Anadyr, Russia | 66.09N, 175.45E | PMN-252 | NRM 20066102 | Cyt*b* | A6 | [GenBank: AB362440] |
|  | Anadyr, Russia | 66.09N, 175.45E | PMN-253 | NRM 20066103 | Cyt*b* | A7 | [GenBank: AB362441] |
|  | Magadan, Russia | 59.18N,　 148.59E | Maga05Pb11 |  | ND5, Cyt*b*, CR-ND6-12S-tRNA | A3 |  |
|  | Magadan, Russia | 59.38N, 150.50E | MagaM1 |  | Cyt*b* | A8 | [GenBank: AB362442] |
|  | Magadan, Russia | 59.38N, 150.50E | MagaM2 |  | Cyt*b* | A9 | [GenBank: AB362443] |
|  | Magadan, Russia | 59.38N, 150.50E | MagaM6 |  | Cyt*b* | A9 |  |
|  | Magadan, Russia | 59.38N, 150.50E | MagaM9 |  | Cyt*b* | A10 | [GenBank: AB362444] |
|  | Magadan, Russia | 59.38N, 150.50E | MagaM10 |  | Cyt*b* | A11 | [GenBank: AB362445] |
|  | Magadan, Russia | 59.38N, 150.50E | MagaM9027 |  | Cyt*b* | A12 | [GenBank: AB362446] |
|  | Arkhangelskaya, Russia | 65.72N, 44.37E | Pborealis747 | UWBM82173 | Cyt*b* | A13 | [GenBank: AB530997] |
|  | Tyumenskaya, Russia | 63.48N, 74.87E | Pborealis3263 | UWBM56562 | Cyt*b* | A14 | [GenBank: AB530998] |
|  | Irkutskaya, Russia | 62.17N, 108.33E | Pborealis2481 | UWBM78398 | Cyt*b* | A15 | [GenBank: AB530999] |
|  | Respublika Tyva, Russia | 50.77N, 91.52E | Pborealis2299 | UWBM66701 | Cyt*b* | A16 | [GenBank: AB531000] |
|  | Respublika Tyva, Russia | 50.77N, 91.52E | Pborealis1912 | UWBM66540 | Cyt*b* | A15 |  |
|  | Arhangay, Mongolia | 48.12N, 100.37E | Pborealis2296 | UWBM58016 | Cyt*b* | A17 | [GenBank: AB531001] |
|  | SE part of Lake Baykal, Russia |  | PborSelenga41 |  | Cyt*b* | A18 | [GenBank: AB531002] |
|  | SE part of Lake Baykal, Russia |  | PborSelenga42 |  | Cyt*b* | A15 |  |
|  | SE part of Lake Baykal, Russia |  | PborSelenga05 |  | Cyt*b* | A19 | [GenBank: AB531003] |
|  | Kamchatka, Russia | 52.11N,　156.37E | Kam-Opara312 |  | Cyt*b* | B2 | [GenBank: AB362425] |
|  | Kamchatka, Russia | 52.11N,　156.37E | Kam-Opara319 |  | ND5, Cyt*b*, CR-ND6-12S-tRNA | B3 | [GenBank: AB362426] |
|  | Kamchatka, Russia | 52.11N,　156.37E | Kam-Opara322 |  | Cyt*b* | B2 |  |
|  | Kamchatka, Russia | 52.11N,　156.37E | Kam-Opara332 |  | Cyt*b* | B4 | [GenBank: AB362427] |
|  | Kamchatka, Russia | 52.11N,　156.37E | Kam-Opara336 |  | Cyt*b* | B5 | [GenBank: AB362428] |
|  | Kamchatka, Russia | 52.11N,　156.37E | Kam-Opara338 |  | Cyt*b* | B2 |  |
|  | Kamchatka, Russia | 52.11N,　156.37E | Kam-Opara344 |  | Cyt*b* | B1 | [GenBank: AB362424] |
|  | Kamchatka, Russia | 52.11N,　156.37E | Kam-Opara345 |  | Cyt*b* | B2 |  |
|  | Kamchatka, Russia | 52.11N,　156.37E | Kam-Opara346 |  | Cyt*b* | B1 |  |
|  | Kamchatka, Russia | 52.11N,　156.37E | Kam-Opara352 |  | Cyt*b* | B6 | [GenBank: AB362429] |
|  | Kamchatka, Russia | 52.11N,　156.37E | Kam-Opara353 |  | Cyt*b* | B2 |  |
|  | Sakhalin, Russia | 52.17N, 141.34E | Sak705 |  | Cyt*b* | B7 |  |
|  | Sakhalin, Russia | 53.10N, 143.12E | Sak04Pb18 |  | Cyt*b* | B10 | [GenBank: AB362433] |
|  | Sakhalin, Russia | 53.10N, 143.12E | Sak04Pb26 |  | Cyt*b* | B11 | [GenBank: AB362434] |
|  | Sakhalin, Russia | 53.10N, 143.12E | Sak04Pb29 |  | Cyt*b* | B9 | [GenBank: AB362432] |
|  | Sakhalin, Russia | 53.10N, 143.12E | Sak04Pb32 |  | Cyt*b* | B1 |  |
|  | Sakhalin, Russia | 53.10N, 143.12E | Sak04Pb42 |  | Cyt*b* | B2 |  |
|  | Hokkaido, Japan | 44.04N, 145.07E | RausuPb1 |  | Cyt*b* | B7 | [GenBank: AB362430] |
|  | Hokkaido, Japan | 44.04N, 145.07E | RausuPb2 | NSMT-A-3956 | Cyt*b* | B3 |  |
|  | Hokkaido, Japan | 43.46N, 144.43E | Shari707 |  | Cyt*b* | B3 |  |
|  | Hokkaido, Japan | 43.46N, 144.43E | Shari709 |  | Cyt*b* | B3 |  |
|  | Hokkaido, Japan | 43.46N, 144.43E | Syari04Pb1 |  | Cyt*b* | B3 |  |
|  | Hokkaido, Japan | 43.46N, 144.43E | Syari04Pb2 | NSMT-A-3978 | Cyt*b* | B8 | [GenBank: AB362431] |
|  | Hokkaido, Japan | 43.46N, 144.43E | Syari04Pb3 |  | Cyt*b* | B3 |  |
|  | Hokkaido, Japan | 43.46N, 144.43E | Syari04Pb4 |  | Cyt*b* | B3 |  |
|  | Hokkaido, Japan | 43.46N, 144.43E | Syari04Pb5 |  | Cyt*b* | B3 |  |
|  | Hokkaido, Japan | 43.46N, 144.43E | Syari04Pb7 |  | Cyt*b* | B3 |  |
|  | Hokkaido, Japan | 43.46N, 144.43E | Syari04Pb8 |  | Cyt*b* | B3 |  |
|  | Hokkaido, Japan | 43.46N, 144.43E | Syari04Pb10 |  | Cyt*b* | B3 |  |
|  | Iwate, Honshu, Japan | 39.56N, 140.51E | Hachi04Pb1 | NSMT-A-3957 | Cyt*b* | C1 | [GenBank: AB362447] |
|  | Iwate, Honshu, Japan | 39.56N, 140.51E | Hachi04Pb2 | NSMT-A-3958 | Cyt*b* | C1 |  |
|  | Iwate, Honshu, Japan | 39.56N, 140.51E | Hachi04Pb3 | NSMT-A-3959 | Cyt*b* | C1 |  |
|  | Iwate, Honshu, Japan | 39.56N, 140.51E | Hachi04Pb4 |  | Cyt*b* | C1 |  |
|  | Iwate, Honshu, Japan | 39.56N, 140.51E | Hachi04Pb5 |  | Cyt*b* | C1 |  |
|  | Iwate, Honshu, Japan | 39.56N, 140.51E | Hachi275 |  | Cyt*b* | C1 |  |
|  | Iwate, Honshu, Japan | 39.56N, 140.51E | Hachi04Pb7 | NSMT-A-3963 | Cyt*b* | C1 |  |
|  | Iwate, Honshu, Japan | 39.56N, 140.51E | Hachi278 |  | Cyt*b* | C1 |  |
|  | Iwate, Honshu, Japan | 39.56N, 140.51E | Hachi280 |  | Cyt*b* | C1 |  |
|  | Iwate, Honshu, Japan | 39.56N, 140.51E | Hachi281 |  | Cyt*b* | C1 |  |
|  | Iwate, Honshu, Japan | 39.56N, 140.51E | Hachi283 |  | Cyt*b* | C1 |  |
|  | Iwate, Honshu, Japan | 39.56N, 140.51E | Hachi287 |  | Cyt*b* | C1 |  |
|  | Iwate, Honshu, Japan | 39.56N, 140.51E | Hachi289 |  | Cyt*b* | C1 |  |
|  | Iwate, Honshu, Japan | 39.56N, 140.51E | Hachi290 |  | Cyt*b* | C1 |  |
|  | Iwate, Honshu, Japan | 39.56N, 140.51E | Hachi294 |  | Cyt*b* | C11 | [GenBank: AB362457] |
|  | Iwate, Honshu, Japan | 39.56N, 140.51E | Hachi295 |  | Cyt*b* | C1 |  |
|  | Iwate, Honshu, Japan | 39.56N, 140.51E | Hachi305 |  | Cyt*b* | C1 |  |
|  | Iwate, Honshu, Japan | 39.56N, 140.51E | Hachi306 |  | Cyt*b* | C1 |  |
|  | Iwate, Honshu, Japan | 39.56N, 140.51E | Hachi307 |  | Cyt*b* | C1 |  |
|  | Iwate, Honshu, Japan | 39.56N, 140.51E | Hachi310 |  | Cyt*b* | C1 |  |
|  | Yamanashi, Honshu, Japan | 36.03N, 138.22E | YatsgLBY |  | Cyt*b* | C5 | [GenBank: AB362451] |
|  | Yamanashi, Honshu, Japan | 36.03N, 138.22E | YatsgWLG |  | Cyt*b* | C1 |  |
|  | Yamanashi, Honshu, Japan | 36.03N, 138.22E | YatsgMM |  | Cyt*b* | C10 | [GenBank: AB362456] |
|  | Yamanashi, Honshu, Japan | 36.03N, 138.22E | YatsgOO |  | Cyt*b* | C1 |  |
|  | Yamanashi, Honshu, Japan | 36.03N, 138.22E | Yatsg701 |  | Cyt*b* | C1 |  |
|  | Yamanashi, Honshu, Japan | 36.03N, 138.22E | Yatsg702 |  | Cyt*b* | C1 |  |
|  | Yamanashi, Honshu, Japan | 36.03N, 138.22E | Yatsg703 |  | Cyt*b* | C6 | [GenBank: AB362452] |
|  | Yamanashi, Honshu, Japan | 36.03N, 138.22E | Yatsg704 |  | Cyt*b* | C2 | [GenBank: AB362448] |
|  | Shizuoka, Honshu, Japan | 35°21'N, 138°46'E | Fuji516 |  | Cyt*b* | C3 | [GenBank: AB362449] |
|  | Shizuoka, Honshu, Japan | 35.21N, 138.46E | Fuji520 |  | Cyt*b* | C1 |  |
|  | Shizuoka, Honshu, Japan | 35.21N, 138.46E | Fuji134 |  | Cyt*b* | C11 |  |
|  | Shizuoka, Honshu, Japan | 35.21N, 138.46E | Fuji534 |  | Cyt*b* | C3 |  |
|  | Shizuoka, Honshu, Japan | 35.21N, 138.46E | Fuji535 |  | Cyt*b* | C11 |  |
|  | Shizuoka, Honshu, Japan | 35.21N, 138.46E | Fuji537 |  | Cyt*b* | C3 |  |
|  | Shizuoka, Honshu, Japan | 35.21N, 138.46E | Fuji515 |  | ND5, Cyt*b*, CR-ND6-12S-tRNA | C4 | [GenBank: AB362450] |
|  | Shizuoka, Honshu, Japan | 35.21N, 138.46E | Fuji545 |  | Cyt*b* | C11 |  |
|  | Shizuoka, Honshu, Japan | 35.21N, 138.46E | Fuji550 |  | Cyt*b* | C1 |  |
|  | Shizuoka, Honshu, Japan | 35.21N, 138.46E | Fuji557 |  | Cyt*b* | C3 |  |
|  | Shizuoka, Honshu, Japan | 35.21N, 138.46E | Fuji567 |  | Cyt*b* | C3 |  |
|  | Nara, Honshu, Japan | 34.12N, 136.05E | odaiPb1 |  | Cyt*b* | C7 | [GenBank: AB362453] |
|  | Nara, Honshu, Japan | 34.12N, 136.05E | odaiPb2 |  | Cyt*b* | C7 |  |
|  | Nara, Honshu, Japan | 34.12N, 136.05E | odaiPb3 |  | Cyt*b* | C9 | [GenBank: AB362455] |
|  | Nara, Honshu, Japan | 34.12N, 136.05E | odaiPb4 |  | Cyt*b* | C9 |  |
|  | Ehime, Shikoku, Japan | 33.46N, 133.7E | ishizuchi801 |  | Cyt*b* | C1 |  |
|  | Ehime, Shikoku, Japan | 33.46N, 133.7E | ishizuchi802 |  | Cyt*b* | C1 |  |
|  | Ehime, Shikoku, Japan | 33.46N, 133.7E | ishizuchi803 |  | Cyt*b* | C11 |  |
|  | Ehime, Shikoku, Japan | 33.46N, 133.7E | ishizuchi804 |  | Cyt*b* | C8 | [GenBank: AB362454] |
|  | Ehime, Shikoku, Japan | 33.46N, 133.7E | ishizuchi805 |  | Cyt*b* | C11 |  |
|  | Oita, Kyushu, Japan | 32.49N, 131.20E | sobo966 |  | Cyt*b* | C12 | [GenBank: AB362458] |
|  | Oita, Kyushu, Japan | 32.49N, 131.20E | sobo967 |  | Cyt*b* | C11 |  |
| *Phylloscopus magnirostris* | Daozhen county, Guizhou, China | 29.10N, 107.34E | | KUNHM 97366 | Cyt*b* |  | [GenBank: XXXXX] |
| *Phylloscopus borealoides* | Iwate, Japan | 39.56N, 140.51E | 1D-36291 |  | ND5, Cyt*b*, CR-ND6-12S-tRNA | | [GenBank: AB362459] |
| *Phylloscopus plumbeitarsus* | Mongolia | 48.24N, 108.01E | Pp29 |  | ND5, Cyt*b*, CR-ND6-12S-tRNA | | [GenBank: AB362468] |
| *Phylloscopus coronatus* | Iwate, Japan | 39.56N, 140.51E | 1D-36293 |  | Cyt*b* |  | [GenBank: AB362460] |

GenBank numbers are given only the first time a haplotype is mentioned. NSMT=National Museum of Nature and Science, Tokyo; NRM= Swedish Museum of Natural History, Stockholm; KUNHM=Kansas University Natural History Museum, Lawrence; UWBM=Burke Museum, University of Washington, Seattle. Cyt*b* = cytochrome *b*.
